# Phase I trial to evaluate the addition of alisertib to fulvestrant in women with endocrine-resistant, ER+ metastatic breast cancer

**DOI:** 10.1007/s10549-017-4616-7

**Published:** 2017-12-30

**Authors:** Tufia C. Haddad, Antonino D’Assoro, Vera Suman, Mateusz Opyrchal, Prema Peethambaram, Minetta C. Liu, Matthew P. Goetz, James N. Ingle

**Affiliations:** 1 Division of Medical Oncology, Mayo Clinic, 200 First Street S.W., Rochester, MN 55905 USA; 2 Department of Biochemistry and Molecular Biology, Mayo Clinic, Rochester, MN USA; 3 Department of Biostatistics, Mayo Clinic, Rochester, MN USA; 4 Division of Oncology, Roswell Park Cancer Center, Buffalo, NY USA; 5 Department of Laboratory Medicine and Pathology, Mayo Clinic, Rochester, MN USA

**Keywords:** Aurora A kinase, Alisertib, Fulvestrant, Breast cancer, Estrogen receptor

## Abstract

**Purpose:**

In estrogen receptor-positive (ER+) breast cancer models, activation of Aurora A kinase (AURKA) is associated with downregulation of ERα expression and resistance to endocrine therapy. Alisertib is an oral selective inhibitor of AURKA. The primary objectives of this phase I trial were to determine the recommended phase II dose (RP2D) and evaluate the toxicities and clinical activity of alisertib combined with fulvestrant in patients with ER+ metastatic breast cancer (MBC).

**Methods:**

In this standard 3 + 3 dose-escalation phase I study, postmenopausal patients with endocrine-resistant, ER+ MBC previously treated with endocrine therapy were assigned to one of two dose levels of alisertib (40 or 50 mg) in combination with fixed-dose fulvestrant.

**Results:**

Ten patients enrolled, of which nine were evaluable for the primary endpoint. The median patient age was 59. All patients had secondary (acquired) endocrine resistance, and all had received prior aromatase inhibitor. Six had experienced disease progression on fulvestrant. There were no severe (grade 3+) toxicities reported during cycle 1 at either dose level. The median progression-free survival time was 12.4 months (95% CI 5.3–not met), and the 6-month clinical benefit rate was 77.8% (95% CI 40.0–87.2%).

**Conclusions:**

In patients with endocrine-resistant, ER+ MBC, alisertib in combination with fulvestrant was well tolerated. A favorable safety profile was observed. The RP2D is 50 mg twice daily on days 1–3, 8–10, and 15–17 of a 28-day cycle with standard dose fulvestrant. Promising antitumor activity was observed, including activity among patients with prior progression on fulvestrant.

## Introduction

Each year, approximately 1.2 million women worldwide are diagnosed with estrogen receptor-positive (ER+) breast cancer [1]. While 5 years of adjuvant endocrine therapy significantly reduces the risk of recurrence, the cumulative risk of a distant recurrence during years 5–14 ranges from 9.8% for N0 (node-negative) disease to 27.7% for N2 (4–9 node-positive) disease [2, 3].

Both de novo and acquired resistance to endocrine therapy remain a major clinical problem [4, 5]. Although most endocrine-resistant breast tumors retain ERα expression, loss of ERα is a well-described mechanism of resistance associated with aggressive tumor behavior and poor clinical outcomes [4–9]. Currently, there are no FDA-approved approaches which reverse endocrine resistance associated with downregulation or loss of ERα expression.

During tumor progression, deregulated activation of Aurora A kinase (AURKA) is functionally linked to epithelial-to-mesenchymal transition (EMT) reprogramming and expansion of a subpopulation of tumor-initiating cells harboring a CD44^+^/CD24^low/−^ phenotype [10–12]. These tumor-initiating cells have stem cell-like properties characterized by their capacity to self-renew, resist drug therapies, and promote distant metastases [13]. In luminal ER+ breast cancer models, activation of AURKA is required to induce EMT and clonal expansion of CD44^+^/CD24^low/−^ cells, thus driving tumor progression [14]. These cells are further characterized by loss of ERα protein expression and resistance to endocrine therapy [15]. Moreover, aberrant AURKA activity is required to induce the expression of SMAD5 and SOX2 [14, 15], two master transcription factors involved in the regulation of EMT and stemness reprogramming [16–19].

In translational studies, residual tumor specimens collected from women with operable ER+ breast cancer following neoadjuvant letrozole contain a significantly enriched CD44^+^/CD24^low/−^ subpopulation and upregulation of mesenchymal genes as compared to their pre-treatment tumor [20]. Kinase inhibitor screens in both endocrine-sensitive and endocrine-resistant cell lines identified AURKA as a potential treatment target in ER+ breast cancer [21, 22]. Moreover, in women with operable ER+ breast cancer treated with tamoxifen, both disease-free and overall survival (OS) were shorter among those with high levels of tumor expression of AURKA [21]. Similar findings from Siggelkow et al. demonstrated that high levels of tumor AURKA expression were associated with decreased metastasis-free survival in women with lymph node-negative breast cancer who had not received adjuvant chemotherapy [23]. Thus, this novel function of AURKA has untapped potential as a biomarker and therapeutic target for endocrine-resistant breast cancer.

In endocrine-resistant, ER+ breast cancer models, alisertib, a selective inhibitor of AURKA, was found to reverse stemness reprogramming and thereby restore the CD44^−^/CD24^+^ phenotype, ERα expression, and sensitivity to endocrine therapy [14, 15]. Moreover, alisertib was found to reduce cellular proliferation in tamoxifen-resistant cells, and this effect was enhanced with the addition of fulvestrant [15]. In summary, based on this preclinical and translational data, we hypothesized that inhibition of AURKA in endocrine-resistant breast cancer could lead to a new therapeutic strategy to restore endocrine sensitivity by targeting the CD44^+^/CD24^low/−^/ERα^low/−^ tumor-initiating cells that promote endocrine resistance.

In the clinical development of alisertib, its safety and tolerability profile has been well defined at the recommended phase II dose (RP2D) of 50 mg tablets orally twice daily on days 1–7 of a 21-day cycle [24, 25]. In a phase II trial of alisertib monotherapy at the RP2D, alisertib was associated with a 6-month clinical benefit rate (CBR = CR + PR + SD for ≥ 6 months) of 54% and median progression-free survival (PFS) of 7.9 months in those with heavily pre-treated, ER+/HER2-negative breast cancer (*n* = 26) [26]. Neutropenia (57%) and leukopenia (36%) were the most frequently reported severe toxicities among the 49 breast cancer patients enrolled [26]. An alternative 28-day, ‘pulse dose’ regimen with alisertib given days 1–3, 8–10, and 15–17 was studied in combination with paclitaxel in a triple-negative breast cancer xenograft model, and it was associated with similar antitumor activity compared with the 7-day continuous schedule at the RP2D [27]. Previous studies modeling hematologic toxicity also predicted that the pulse dose regimen would decrease the incidence of dose-limiting neutropenia compared with a 7-day continuous schedule [28].

The primary objectives of this phase I trial were to evaluate the toxicities and clinical activity of alisertib with fulvestrant in patients with ER+ advanced breast cancer. The ‘pulse dose’ schedule of alisertib was pursued as its 28-day schedule was compatible with the standard 28-day schedule of fulvestrant.

## Methods

### Patients

Eligible patients were postmenopausal women age ≥ 18 years who had histologically confirmed metastatic or locally advanced, unresectable breast cancer that was estrogen and/or progesterone receptor positive and had progressed on at least one prior line of endocrine therapy. Unlimited prior endocrine therapies were allowed. Prior fulvestrant was not mandated. One prior line of chemotherapy was required in either the (neo)adjuvant or metastatic setting, and no more than 2 prior lines of chemotherapy were allowed in the metastatic setting. An Eastern Cooperative Oncology Group (ECOG) performance status ≤ 1, life expectancy of ≥ 4 months, and adequate hematologic, hepatic, and renal function were required. Measurable or non-measurable disease per RECIST criteria (v.1.1) and stable treated CNS metastases were allowed. Patients necessitating routine use of proton pump inhibitors, H2-blockers, or pancreatic enzymes were ineligible as concurrent administration of these medications with alisertib has been associated with increased alisertib exposure. This study was performed after approval by the Mayo Institutional Review Board in accordance with assurances filed with and approved by the Department of Health and Human Services. All patients provided written informed consent.

### Study design

A 3 + 3 phase I clinical trial was conducted to determine if the previously established 50 mg twice daily RP2D of alisertib (on a 21-day schedule) would be tolerable when administered on the ‘pulse dose’ 28-day schedule in combination with standard dose fulvestrant. This schedule was selected as it was deemed compatible with the rigid 28-day schedule of fulvestrant. Alisertib was administered twice daily by mouth on days 1–3, 8–10, and 15–17 of each cycle. Two dose levels were planned for evaluation (40 and 50 mg) as previous studies with the 21-day schedule showed substantial toxicity above the 50 mg dose level. The starting dose level was 40 mg. Fulvestrant was given as 500 mg IM on days 1 & 15 of cycle 1 and day 1 of all subsequent cycles. Adverse events and safety laboratory studies were recorded at the end of each treatment cycle. As hair loss has been associated with alisertib, it was also self-assessed at the end of each treatment cycle using the Modified WHO scale [29]. Tumor assessments occurred after every 2 cycles of therapy.

### Statistical analysis

The decision to dose escalate/de-escalate was based on the number and severity of the toxicities that developed during the first cycle of treatment.

Dose-limiting toxicities (DLT) included the following: febrile neutropenia with grade ≥ 3 neutropenia; grade ≥ 4 anemia; grade 3 neutropenia lasting 5 or more days; grade ≥ 4 neutropenia; grade 3 thrombocytopenia with grade ≥ 3 bleeding; grade ≥ 4 thrombocytopenia; grade ≥ 3 acute kidney injury, somnolence, oral mucositis, or other non-hematologic toxicity; and a delay in treatment of greater than 2 weeks due to toxicity.

If 2 or more of the 6 patients treated at the 40 mg dose develop a DLT, the next cohort of 3 patients was to be enrolled at the 30 mg dose level. If at most 0 out of 3 patients or 1 out of 6 patients developed a DLT at the 40 mg, the next cohort of 3 patients was enrolled at the 50 mg dose level. No other dose levels were considered.

### Study endpoints and assessments

All patients meeting the eligibility criteria who provided written informed consent and began protocol-directed therapy were included in the analysis of the safety and clinical outcome data. The data lock for this report was October 23, 2017.

The primary endpoint of this trial was to determine if the previously established RP2D of alisertib (50 mg) on 21-day schedule was tolerable when administered on a ‘pulse dose’ 28-day schedule in combination with standard dose of fulvestrant. The RP2D of alisertib in combination with fulvestrant was defined as the highest dose level, among those under consideration (up to 50 mg), where at most 1 of 6 patients develops a DLT.

Tumor response was defined by RECIST criteria (v. 1.1) for a partial or complete response (PR or CR) on two consecutive evaluations at least 8 weeks apart. Duration of tumor response was the time from registration to disease progression. PFS was the time from registration to documentation of the first of the following events: local, regional, or distant recurrence, diagnosis of contralateral breast disease, diagnosis of a second primary, or death due to any cause. Survival time was the time from registration to death due to any cause. The distribution of event times was estimated using the Kaplan–Meier method.

### Correlative studies

Archived tumor tissue samples from either the primary tumor or a metastatic site were available for 7 of the 9 evaluable subjects. These tissue samples will be used for analysis of ERα, phosphorylated (p) pAURKA, pSMAD5, and pSOX2 expression. This work is ongoing in support of the follow-up phase II study.

## Results

### Study population

From September 8, 2014 to April 2, 2015, 10 women were enrolled (3 at 40 mg dose and 7 at 50 mg dose). One patient entered at the 50 mg dose level was found to be ineligible and replaced as she was on a proton pump inhibitor at the time of registration. Patient and tumor baseline characteristics of the remaining nine patients are presented in Table 1.

**Table 1:** Patient baseline characteristics

Baseline characteristics	Evaluable patients *N* = 9
Median age (range)	59 years (48–73 years)
ECOG performance status	
0	4 (44.4%)
1	5 (55.5%)
Histology	
Ductal	6 (66.7%)
Lobular	3 (33.3%)
Specimen used for ER, PR, and HER2 testing collected at	
Primary diagnosis	3 (33.3%)
Previous metastatic episode	5 (55.6%)
Current metastatic episode	1 (11.1%)
Biomarker status	
ER-positive	9 (100%)
PR-positive	9 (100%)
HER2-negative	9 (100%)
Metastatic relapse on or within 1-year completion of adjuvant endocrine therapy	
Yes	5 (55.6%)
No	4 (44.4%)
Lines of hormonal therapy in the metastatic setting	
0	1 (11.1%)
1	2 (22.2%)
2	2 (22.2%)
3 or more	4 (44.4%)
Prior hormonal therapies in the metastatic setting	
Non-steroidal AI	8 (88.9%)
Fulvestrant	6 (66.7%)
Exemestane + everolimus	5 (55.6%)
Tamoxifen	2 (22.2%)
Z-Endoxifen	2 (22.2%)
Lines of chemotherapy in metastatic setting	
0	3 (33.3%)
1	4 (44.4%)
2	2 (22.2%)
Hair loss	
None	6 (66.7%)
≤ 10%	2 (22.2%)
> 75%	1 (11.1%)
Pre-treatment toxicities	
Grade 1 anemia	3 (33.3%)
Grade 1 fatigue	3 (33.3%)
Grade 1 neutropenia	1 (11.1%)
Grade 1 kidney injury	1 (11.1%)

The median patient age was 59 (range 48–73 years). All patients had secondary endocrine resistance defined by the ESO-ESMO guidelines as recurrence on but after the first 2 years of adjuvant endocrine therapy, or recurrence within 12 months of completing adjuvant endocrine therapy, or metastatic disease progression occurring on but ≥ 6 months after initiating first-line endocrine therapy [30]. Prior endocrine treatments included an aromatase inhibitor (100%), fulvestrant (66.7%), tamoxifen (55.6%), and everolimus-based endocrine regimen (55.6%). Four patients had received anthracycline and taxane-based chemotherapy in the adjuvant setting, and six patients (66.7%) had received chemotherapy in the metastatic setting. Six patients (67%) had visceral metastatic disease, and three patients (33%) had bone-only metastases. Three patients (33%) had measurable disease per RECIST criteria (v. 1.1).

### Dose escalation and RP2D determination

Three patients enrolled onto the 40 mg dose level of alisertib and completed their first cycle of treatment without developing a DLT. Thus, the next cohort of 3 patients was enrolled onto the 50 mg dose level of alisertib. None of these 3 patients developed a DLT during cycle 1, so an additional 3 patients were enrolled onto the 50 mg dose level of alisertib. As none of the 6 patients enrolled onto the 50 mg dose level of alisertib developed a DLT during their first cycle of treatment, 50 mg twice daily of alisertib is the RP2D on a 28-day schedule when used in combination with standard dosing of fulvestrant.

### Safety

Grade 2 toxicities reported during the first cycle of treatment included: neutropenia (44.4%), leukopenia (22.2%), anemia (11.1%), diarrhea (11.1%), and alopecia (11.1%). There were no grade 3 or 4 toxicities reported during cycle 1. The toxicities reported across all cycles of treatment regardless of attribution are presented in Table 2.

**Table 2:** Grade ≥ 2 adverse events during all treatment cycles regardless of attribution

Toxicity	Grade		
	2	3	4
Hematologic adverse events			
Anemia	2 (22.2%)	0	0
Leukopenia	1 (11.1%)	2 (22.2%)	0
Lymphopenia	1 (11.1%)	1 (11.1%)	0
Neutropenia	2 (22.2%)	0	2 (22.2%)
Non-hematologic adverse events			
Alopecia	4 (44.4%)	0	0
Anxiety	1 (11.1%)	0	0
Depression	1 (11.1%)	0	0
Diarrhea	2 (22.2%)	0	0
Dry mouth	1 (11.1%)	0	0
Dyspnea	0	1 (11.1%)	0
Fatigue	2 (22.2%)	1 (11.1%)	0
Hypertension	0	0	1 (11.1%)
Hypoxia	0	1 (11.1%)	0
Insomnia	1 (11.1%)	0	0
Nausea	1 (11.1%)	0	0
Nervous system disorder-leg cramp	1 (11.1%)	0	0
Oral mucositis	1 (11.1%)	0	0
Tremor	1 (11.1%)	0	0

Three patients, all treated at the 50 mg dose of alisertib, experienced severe toxicities during the course of treatment. Two patients presented with treatment-related grade 4 neutropenia with grade 3 leukopenia. One patient presented with grade 4 hypertension and grade 3 hypoxia and dyspnea, all considered unlikely related to treatment. There was limited evidence of cumulative toxicity in the most frequently observed adverse events.

With regard to alopecia, one patient had complete hair loss at the time of enrollment, and she experienced regrowth of her hair during therapy. Each of the 6 patients who had no hair loss at study entry experienced hair loss with treatment: ≤ 10% (1 patient), 11–30% (1 patient), 31–75% (2 patients), and > 75% (2 patients). Two patients with ≤ 10% hair loss at study entry had 31–75% and > 75% hair loss with treatment, respectively.

### Clinical activity

Three of the 9 evaluable patients enrolled had measurable disease. They included a 61-year-old woman with lobular ER+/PR+/HER2-breast cancer that had metastasized to the liver and bone who enrolled at the 50 mg dose level and had a partial response lasting 11.1 months; a 53-year-old woman with lobular ER+/PR+/HER2-breast cancer that had metastasized to the bone, distant nodes, abdomen, and ovary who enrolled at the 50 mg dose level and had a partial response lasting 25.9 months; and a 59-year-old woman with ductal ER+/PR+/HER2-breast cancer that had metastasized to the bone, distant nodes, abdomen, liver, and lung who enrolled at the 40 mg dose level who had stable disease lasting 3.8 months.

**Fig. 1:**
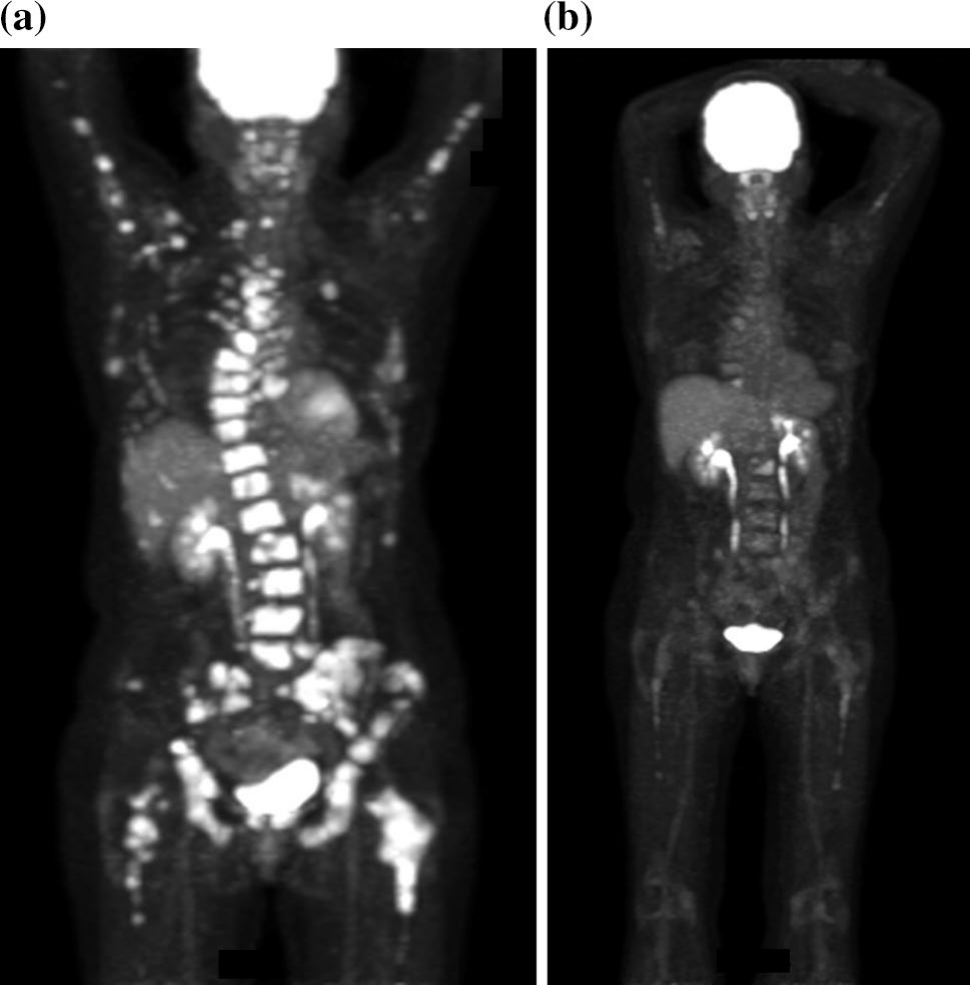
Antitumor activity of alisertib and fulvestrant in a patient with prior progression during five different lines of endocrine therapy for metastatic disease, including tamoxifen, letrozole, fulvestrant, exemestane plus everolimus, and Z-endoxifen (on phase I trial): **a** baseline before starting alisertib and fulvestrant, and **b** after 2 cycles of treatment

There were 6 patients with non-measurable disease. One of 2 patients with non-measurable disease enrolled at the 40 mg dose level and all 4 patients with non-measurable disease enrolled at the 50 mg dose level maintained stable disease at least 6 months. Notably, a 51-year-old woman with fulvestrant-resistant, ER+/PR+/HER2-breast cancer that had metastasized to the bone treated at the 40 mg dose level experienced a dramatic decline in FDG activity of her disease by PET CT imaging after 2 cycles of treatment (Fig. 1). This was further corroborated by a decline in her CA 15-3 tumor marker from 821 to 126 U/mL, and she maintained stable disease for 13.8 months.

Thus, the 6-month CBR was 77.8% (95% CI 40.0–87.2%).

### Clinical outcome

**Fig. 2:**
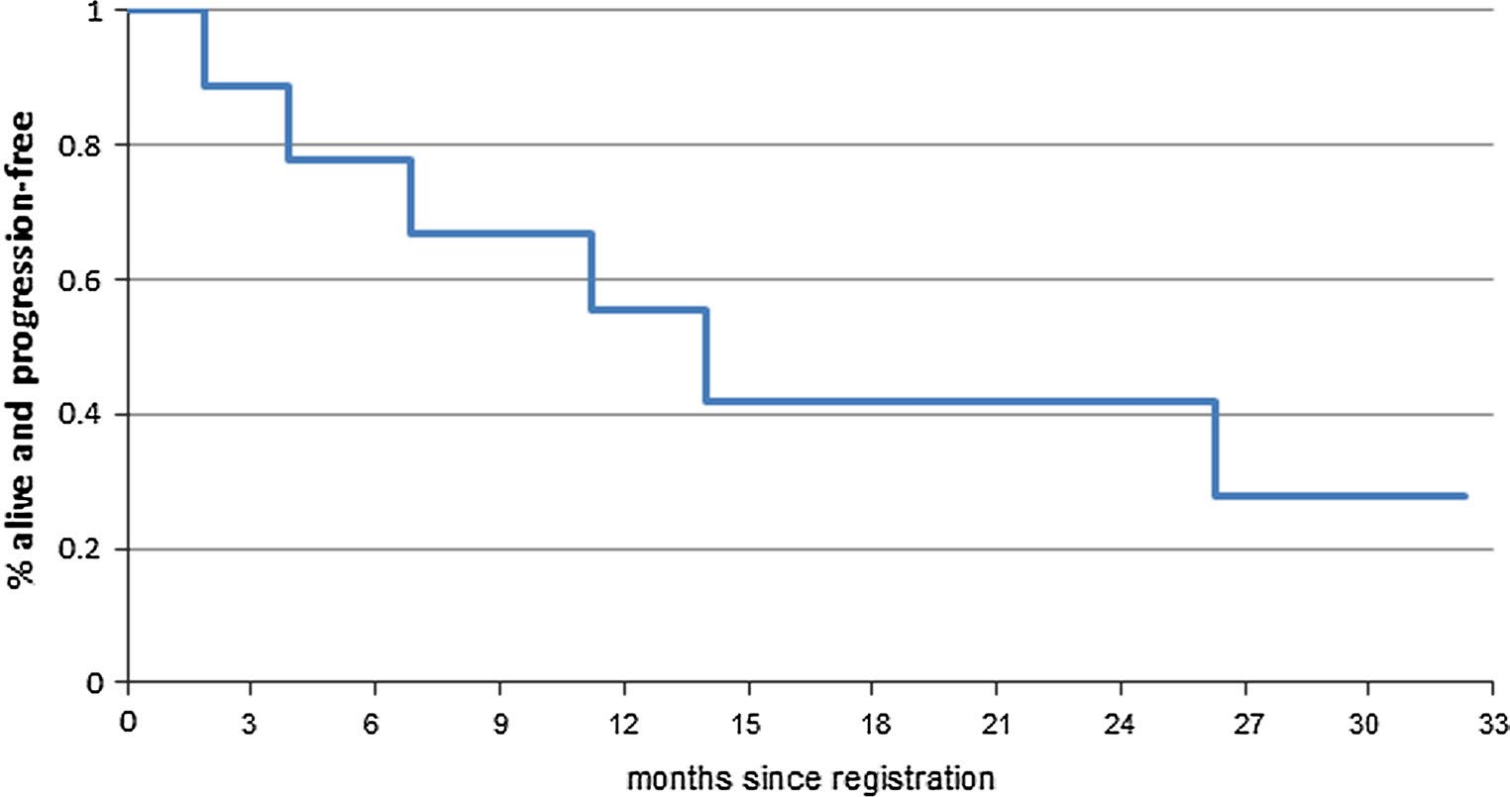
Progression-free survival distribution

There is one patient enrolled on 50 mg dose level who continues on study treatment with stable disease at cycle 35 (31.2 months). Another patient enrolled on 50 mg dose level discontinued study treatment after 14 cycles due to grade 4 hypertension with grade 3 dyspnea and hypoxia (all considered unlikely related to treatment); she remains alive without disease progression on fulvestrant. The remaining 7 patients have all discontinued treatment due to disease progression, and three of these patients have subsequently died. The one-year PFS rate is 55.6% (95% CI 31.0–99.7%) with a median PFS time of 12.4 months (95% CI 5.3–not met) (Fig. 2). The treatment course, grade 3 or higher toxicities, and clinical outcomes of each of the 9 evaluable patients are summarized by dose level in Table 3.

**Table 3:** Treatment course and outcome

Alisertib dose level	Patient	≥ grade 3 toxicity, all cycles	Progression-free survival (months)	Overall survival (months)
40 mg	1	None	3.8	19.2
	2	None	13.8	31.5
	3	None	32.5	32.5+
50 mg	1	None	1.8	6.4
	2	Grade 4 neutropenia with grade 3 leukopenia and lymphopenia	6.7	7.6+
	3	None	11.1	11.7+
	4	Grade 3 fatigue and then grade 4 hypertension with grade 3 dyspnea and hypoxia	13.3+^a^	13.3+
	5	None	25.9	27.6
	6	Grade 4 neutropenia with grade 3 leukopenia	31.8+	31.8+

^a^ One subject discontinued study participation due to grade 4 hypertension with grade 3 dyspnea and hypoxia (considered unlikely related to treatment) after 13.3 months of therapy. As of the data lock, the patient had maintained stable disease on fulvestrant monotherapy

## Discussion

Given the previously observed promising single-agent activity of alisertib in heavily pre-treated, endocrine-resistant advanced breast cancer [26] and preclinical studies demonstrating enhanced antitumor activity when combined with fulvestrant [14, 15], we performed a phase I study that demonstrated alisertib 50 mg twice daily on days 1–3, 8–10, and 15–17 of a 28-day cycle in combination with fulvestrant was clearly tolerable, and it is the RP2D when this alisertib schedule is utilized with fulvestrant for evaluation in future trials. We did not pursue higher alisertib dose levels as previous studies with the 21-day schedule showed substantial toxicity above the 50 mg dose level.

Hematologic toxicity grade ≥ 3 was not observed during cycle one. Across all cycles, it was limited to two grade 3 leukopenia and two grade 4 neutropenia events in two patients (*n* = 2, 22%). Grade 1–2 anemia was common; however, there were no grade ≥ 3 events. Grade 3 gastrointestinal toxicities were not observed, and low-grade nausea and vomiting were uncommon. Importantly, there was only a single episode of grade 2 oral mucositis (*n* = 1, 11%) and no grade ≥ 3 events. These findings are notable given that stomatitis and neutropenia were common DLTs during alisertib development. Specifically, in breast cancer patients receiving alisertib monotherapy (50 mg dose on days 1–7 of a 21-day cycle), the grade 1–2 stomatitis event rate was 30%, grade 3–4 stomatitis event rate was 15%, and grade 3–4 neutropenia rate was 57%. The pulse dose, 28-day schedule of alisertib utilized in this trial resulted in significantly lower grade ≥ 3 event rates relative to those observed in prior studies with the traditional 21-day cycle of alisertib. There was limited evidence of cumulative toxicity in the most frequently observed adverse events.

Striking clinical activity was observed for the combination of alisertib and fulvestrant in patients with secondary endocrine resistance. The 6-month CBR of 78% is an important finding given that all patients had received prior aromatase inhibitor and two-thirds prior fulvestrant. These data provide support to the working hypothesis that alisertib restores endocrine sensitivity. The objective response rate to this combination remains to be defined due to the fact the majority of the patients had non-measurable disease at enrollment; it is notable, however, that 2 of 3 patients with measurable disease had a partial response to therapy.

The median PFS was 12.4 months. Six (67%) patients received ≥ 12 cycles of therapy, including one patient who remains on active treatment after 31 months. This PFS time is longer than the median PFS of 7.9 months observed in a similar patient population (ER+, endocrine-resistant) receiving alisertib monotherapy [26]. Both of these median PFS times exceed what has been typically observed in second-line endocrine therapy trials in which the median PFS has varied between 2.8 and 4.6 months [31, 32]; however, it is noteworthy that in a similar patient population treated in MONARCH-2, median PFS for the fulvestrant/placebo arm was 9.3 months [33]. Nonetheless, both CBR and PFS times are in the range of recently FDA-approved combinations of targeted agents with endocrine therapy [32, 34, 35]. As such, these promising efficacy results and the favorable toxicity profile observed in this study have led to the development and activation of a phase II evaluation of alisertib alone and in combination with fulvestrant in endocrine-resistant metastatic breast cancer (NCT02860000; TBCRC041).

Despite many strengths of this trial, the authors acknowledge its limitations. The study sample size is small. While sufficient to address the primary endpoint, the substantial efficacy and tolerability of this regimen observed in our study need to be confirmed in subsequent clinical trials powered for survival endpoints. In addition to this, pharmacokinetic studies were not incorporated into the study design. Given that lower rates of well-established dose-limiting toxicities were observed in this trial with the pulse dose alisertib regimen, it is feasible that patients may have had lower drug levels enabling the more favorable toxicity profile. It is also feasible that there was a drug interaction between fulvestrant and alisertib facilitating exposure to lower alisertib concentrations. Finally, while archived tumor biospecimens were collected from 7 of 9 patients, a mix of primary and metastatic tumors were retrieved. Given this heterogeneity and the small and incomplete sample size, the correlative biomarker results were not included in this manuscript.

In conclusion, the combination of alisertib and fulvestrant is tolerable with limited grade ≥ 3 adverse events relative to what has been observed in prior phase II studies with alisertib. In addition to this, the regimen appears to be highly effective even among those with secondary endocrine resistance and previously treated with chemotherapy. The clinical activity observed when the majority of patients had prior progression on fulvestrant is consistent with preclinical data that suggest alisertib can target endocrine-resistant ER^low/−^ breast tumor-initiating cells and restore endocrine sensitivity. Further evaluation of alisertib in combination with fulvestrant is warranted and indeed ongoing.
